# Novel Designed Proteolytically Resistant VEGF-B186R127S Promotes Angiogenesis in Mouse Heart by Recruiting Endothelial Progenitor Cells

**DOI:** 10.3389/fbioe.2022.907538

**Published:** 2022-08-04

**Authors:** Rahul Mallick, Erika Gurzeler, Pyry I. Toivanen, Tiina Nieminen, Seppo Ylä-Herttuala

**Affiliations:** ^1^ A.I.Virtanen Institute for Molecular Sciences, University of Eastern Finland, Kuopio, Finland; ^2^ Kuopio Center for Gene and Cell Therapy, Kuopio, Finland; ^3^ Heart Center and Gene Therapy Unit, Kuopio University Hospital, Kuopio, Finland

**Keywords:** angiogenesis, endothelial progenitor cell (EPC), gene therapy, inflammation, VEGF-B

## Abstract

**Background:** Previous studies have indicated that vascular endothelial growth factor B186 (VEGF-B186) supports coronary vascular growth in normal and ischemic myocardium. However, previous studies also indicated that induction of ventricular arrhythmias is a severe side effect preventing the use of VEGF-B186 in cardiac gene therapy, possibly mediated by binding to neuropilin 1 (NRP1). We have designed a novel VEGF-B186 variant, VEGF-B186R127S, which is resistant to proteolytic processing and unable to bind to NRP1. Here, we studied its effects on mouse heart to explore the mechanism of VEGF-B186-induced vascular growth along with its effects on cardiac performance.

**Methods:** Following the characterization of VEGF-B186R127S, we performed ultrasound-guided adenoviral VEGF-B186R127S gene transfers into the murine heart. Vascular growth and heart functions were analyzed using immunohistochemistry, RT-PCR, electrocardiogram and ultrasound examinations. Endothelial progenitor cells (EPCs) were isolated from the circulating blood and characterized. Also, *in vitro* experiments were carried out in cardiac endothelial cells with adenoviral vectors.

**Results:** The proteolytically resistant VEGF-B186R127S significantly induced vascular growth in mouse heart. Interestingly, VEGF-B186R127S gene transfer increased the number of circulating EPCs that secreted VEGF-A. Other proangiogenic factors were also present in plasma and heart tissue after the VEGF-B186R127S gene transfer. Importantly, VEGF-B186R127S gene transfer did not cause any side effects, such as arrhythmias.

**Conclusion:** VEGF-B186R127S induces vascular growth in mouse heart by recruiting EPCs. VEGF-B186R127S is a novel therapeutic agent for cardiac therapeutic angiogenesis to rescue myocardial tissue after an ischemic insult.

## Graphical Abstract

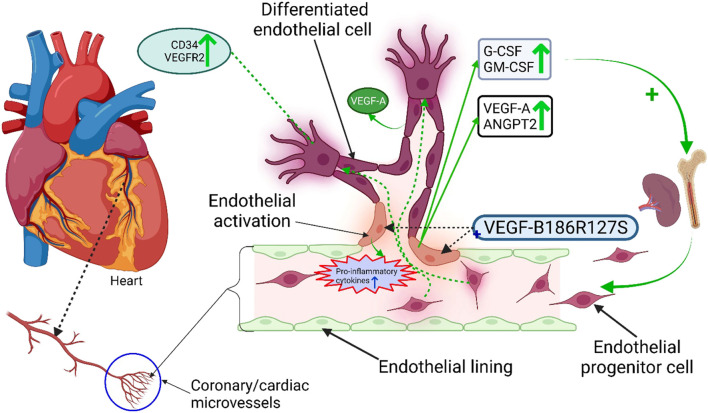

## Introduction

Myocardial ischemia is the most common clinical consequence of cardiovascular disease (Khan et al., 2020). Ischemic changes are associated with a reduction of blood supply to the affected region(s) of the heart (Kontos et al., 2010). It is also known that the subendocardial area is prone to ischemic insults (Algranati et al., 2011). Conventional bypass surgery or catheter-mediated interventions are commonly used to treat occluded coronary vessels along with medication (Ylä-Herttuala et al., 2017). It is noteworthy that despite these reperfusion treatments, subendocardial blood supply is commonly insufficient (Kloner et al., 2018). Therefore, novel therapeutic strategies for promoting capillary and collateral vessel growth are in high demand. Vascular endothelial growth factor A (VEGF-A) is the most commonly studied proangiogenic factor for possible therapy (Ylä-Herttuala and Baker, 2017). VEGF-A binds to vascular endothelial growth factor receptor 1 (VEGFR1) and VEGFR2 to regulate developmental and adaptive vascular growth in the heart (Ferrara et al., 2003). Despite significant angiogenic capability, VEGF-A has failed to meet expectations to treat myocardial ischemia due to vascular leakage and recruitment of inflammatory cells (Ylä-Herttuala and Baker, 2017). Therefore, focus needs to be shifted to other factors with proangiogenic activity.

VEGF-B is highly expressed in metabolically active tissues and known to bind to VEGFR1 and neuropilin 1 (NRP1) (Bry et al., 2014). So far, two alternatively spliced VEGF-B isoforms have been identified: VEGF-B167 (42 kDa), which binds to heparan sulfate proteoglycans in the extracellular matrix and sarcolemma due to a heparin-binding carboxy terminus, and VEGF-B186 (60 kDa), which contains a hydrophobic carboxy-terminus and is proteolytically processed following biosynthesis (Khan et al., 2020). Full-length VEGF-B186 binds to VEGFR1, while proteolytically processed VEGF-B186 N-terminal domain (named as VEGF-B127 due to processing site at Arg-127) binds to both VEGFR1 and NRP1 (Makinen et al., 1999; Bry et al., 2014).

Following acute myocardial infarction, plasma VEGF-B levels are increased, while VEGF-B expression is downregulated during post-myocardial infarction remodeling and heart failure (Devaux et al., 2012; Kivelä et al., 2014). VEGF-B has been shown to improve cardiac contractility in rodent models of myocardial infarction and to induce anti-apoptotic effects in cardiomyocytes (Zentilin et al., 2010). VEGF-B has also been shown to promote coronary vessel growth and capillary enlargement in the heart without increasing permeability, along with improvement of vital cardiac metabolic functions, for example, fatty acid transport and utilization in rodent and pig models (Karpanen et al., 2008; Lähteenvuo et al., 2009; Bry et al., 2010; Serpi et al., 2011; Kivelä et al., 2014). VEGF-B over-expressing animals have increased vessel density and heart size leading to nonpathological cardiac hypertrophy (Lähteenvuo et al., 2009; Kivelä et al., 2014). In addition, there is no significant functional difference between the hearts of transgenic VEGF-B167 or VEGF-B186 mice (Lähteenvuo et al., 2009). Unfortunately, VEGF-B186 gene therapy has been linked with severe arrhythmias (Lähteenvuo et al., 2020), and it has been speculated that VEGF-B186 binding to NRP1 causes sympathetic nerve growth to provoke arrhythmias. To avoid this problem, we recently designed different isoforms of VEGF-B186, as it was unclear whether the angiogenic potential or arrhythmogenic stimuli of VEGF-B186 is due to the full-length VEGF-B186 or whether NRP1 binding after the proteolytic processing contributes to these effects. We demonstrated that full-length VEGF-B186 is solely responsible for angiogenesis, and no arrhythmogenic stimuli were observed with the full length or the NRP1 binding isoforms in this study (Korpela et al., 2021).

However, the mechanisms behind VEGF-B186-induced vascular growth in the heart and the crosstalk between endothelial cells and cardiomyocytes or other cardiac resident cells are still unclear. The aim of this study was to investigate the mechanisms behind the VEGF-B186-induced myocardial vascular growth and explore the role of NRP1 in this process.

## Materials and Methods

### Adenoviral Vector Transduction

Human serotype 5 adenoviral vectors were produced by National Virus Vector Laboratory at Biocenter Kuopio, Finland (Korpela et al., 2021). Adenoviral vector titers were spectrophotometrically determined at OD260 nm (Sweeney and Hennessey, 2002). HeLa cells and human cardiac microvascular endothelial cells (HMVEC-Cs) were used for adenoviral vector transductions. HeLa cells were cultured in Dulbecco’s Modified Eagle’s Medium (DMEM) (Sigma, D6429-500 ML) supplemented with 10% FBS and 1% penicillin–streptomycin. HMVEC-Cs were cultured according to the manufacturer’s protocol (Lonza, United States). In brief, the cells were cultured in EBM^TM^-2 Basal Medium (Lonza, CC-3156) supplemented with EGM^TM^-2 MV Microvascular Endothelial Cell Growth Medium SingleQuots^TM^ (Lonza, CC-4147). The cells used were between passages 3–6. Following seeding the cells into 6-well plates, cells were incubated overnight in a humidified atmosphere with 5% CO2 at 37°C in the corresponding cell line growth medium. The next day, cells were transduced with 1,000 viral particles (vp)/cell, except 4000 vp/cell was used for the C-terminal constructs for the immunoblotting study. 24 h later, cells were washed with PBS, and a fresh cell growth medium was added.

### Immunoblotting

After electrophoresis, proteins were transferred to nitrocellulose membranes (Bio-rad, 1704159) and blocked for 1 h with 5% skimmed milk in TBST (0.1% Tween 20 in TBS). Primary antibodies [anti-VEGF-B (R&D systems, MAB3372) and anti-VEGF-A (Abcam, ab52917)] were incubated overnight at 4°C. After washes, membranes were incubated with secondary peroxidase coupled antibodies [anti-mouse (R&D systems, HAF018) and anti-rabbit (Invitrogen, 31460)] for 1 h at RT. Target proteins were detected using ECL western blot detection solution (Thermo Scientific, 32132).

### Pull-Down Assay

VEGF-B, VEGF-A165, or CMV (control)–containing media from adenoviral vector transduced HeLa cells was pre-cleared with Pierce Protein A/G Magnetic Beads (Thermo Scientific, 88802) and then combined with Fc-tagged soluble hVEGFR1 (R&D systems, 321-FL-050/CF), hNRP1 (R&D systems, 10455-N1-050), or hNRP2 (R&D systems, 2215-N2-025) recombinant proteins and incubated overnight at 4°C. The protein complexes were pulled down with Pierce Protein A/G Magnetic Beads, washed, and eluted with Laemmli sample buffer (containing 10% 2-Mercaptoethanol) at 95°C for 5 min, followed by polyacrylamide gel electrophoresis and immunoblotting.

### Cell Viability and Proliferation Assay

The BaF3-VEGFR1 cell line was used to study the biological activity of the different VEGF-B variants. BaF3-VEGFR1 is a previously described IL-3–dependent mouse pro-B cell line stably expressing human VEGFR1 ectodomain (Landgren et al., 1998; Anisimov et al., 2013). In addition, BaF3-VEGFR1 cells were cultured in DMEM supplemented with 10% FBS, 1% penicillin–streptomycin, 1.25 ml Zeocin (Invitrogen, R25001), and 2 ng/ml rmIL3 (Merck, 407631-M). The assay was performed by plating BaF3-VEGFR1 cells in a culture medium without rmIL3 at 18,000 cells per well and adding VEGF-B186, VEGF-B186R127S, VEGF-A165, or CMV (control)–containing media from adenoviral vector transduced HeLa cells. After 48 h, proliferation and cell viability were measured using Cell titer 96® AQueous One Solution Assay System (Promega, G3582) (Toivanen et al., 2017).

### Mouse Models

For the experiments, a total of 34 specific pathogen-free 12-week-old C57BL/6JOlaHsd male mice aged under a standard chow diet were used. The mice were kept in the National Laboratory Animal Center of The University of Eastern Finland, Kuopio, Finland. The mice were housed under a 12 light/12 dark cycle at a temperature of 22 ± 2°C with 50 ± 10% humidity. All animal procedures were approved by the National Animal Experimental Board of Finland and carried out in accordance with the guidelines of The Finnish Act on Animal Experimentation.

### Ultrasound-Guided Closed-Chest Myocardial Injections

Mice were anesthetized with isoflurane (Isoflurane-Vet 100% w/w, Boehringer Ingelheim, United Kingdom) (induction with 4.5% isoflurane, 450 ml air: maintenance with 1.5% isoflurane, 200 ml air). Ultrasound imaging was performed using a high-resolution imaging system specially developed for small animal research (Vevo 2100, Fujifilm VisualSonics Inc.) before gene transfer on the injection day (d0) and sixth post-injection day (d6). A high-frequency microscan transducer (MS400, Fujifilm VisualSonics Inc.) operating at 18–38 MHz, with a focal depth of 12.7 mm, was used. 0.9% saline or adenoviral vectors were injected into the anterior wall of the left ventricle under ultrasound guidance (Huusko et al., 2010). Study groups are described in Supplementary Table S1. Viral constructs were diluted in 0.9% saline to a final concentration of 1 × 10^12^ vp/ml, and a total of 1 × 10^10^ vp in 10 µL was injected into the mouse myocardium. The injections were carried out under anesthesia with the 30 G × 1″ disposable needle in a 50 µL Hamilton syringe, which was connected to a micromanipulator (Fujifilm VisualSonics Inc.), as described by Springer et al. (2005). Analgesia (carprofen 50 mg/ml, Rimadyl, Pfizer Inc.) was given following the operation.

### Echocardiographic and Electrocardiographic Data Analyses

Echocardiographic data from mice on d0 and d6 were analyzed by using Vevo Lab Sofware, version 5.6.1 (Fujifilm VisualSonics Inc.) in a blinded manner. Measurements were averaged from at least five different cycles (Huusko et al., 2010).

Left ventricular ejection fraction was calculated by Vevo Lab Sofware using the Teicholz formula:

Left ventricular ejection fraction = Stroke volume/Left ventricular end diastolic volume × 100% = (Left ventricular end diastolic volume–Left ventricular systolic volume)/Left ventricular end diastolic volume × 100%.

Left ventricular stroke volume = Left ventricular end diastolic volume–Left ventricular systolic volume.

Left Ventricular Fractional Shortening = (Left ventricular end diastolic diameter—left ventricular end systolic diameter)/Left ventricular end diastolic diameter × 100%

Left ventricular endocardial fractional area change = (Left ventricular end diastolic area–left ventricular end systolic area)/Left ventricular end diastolic area × 100%

Left ventricular mass (corrected) = 0.8 (1.053 × [(Average diastolic diameter at the outer wall)^3^—(Average diastolic diameter at the inner wall)^3^].

Electrocardiographic signals were monitored and recorded from the electrode pads on the mouse platform during an echocardiogram. Electrocardiographic data were analyzed by Kubios HRV analysis program version 3.4.3 0, Kubios Oy, Kuopio, Finland (Merentie et al., 2015).

### Blood Collection and Differential Count

100 µL of blood was collected from the lateral saphenous vein of conscious mice using a restraint tube 48 h before operation. In brief, the hind leg was immobilized in the extended position by applying gentle downward pressure immediately above the knee joint to collect the blood in an aseptic condition. Blood was collected by capillary action into a hematocrit tube containing ethylenediaminetetraacetic acid (EDTA). Following euthanizing by CO_2_ inhalation, blood was collected through cardiac puncture using a 25G needle through the left ventricle. The differential blood count from fresh blood was performed by MOVET Oy, Finland.

### Histological Analyses

Collected murine hearts were perfused with 1% paraformaldehyde (PFA) in PBS (pH 7.4) and immersion fixed in 4% PFA overnight and embedded in paraffin. 5 µm coronal sections were used for histological analyses. Hematoxylin/eosin staining was used to characterize tissue morphology and evaluate injection localization. Immunohistochemical stainings were performed to evaluate microvascular area growth, activated endothelial cells, and the count of recruited immune cells by using CD31 (1:200, BD pharminogen, 550274), CD34 (1:200, Bio-Rad, MCA 1825), and Ly6G (1:200, Santa Cruz Biotechnology, sc-53515) antibodies, respectively (Huusko et al., 2010). DAB chromogen (Vector Laboratories, SK-4100) was used to visualize the staining, and Methyl Green (DAKO, S1952) or Hematoxilyn was used for counterstaining. Images of the sections were taken with a Nikon H550L microscope. Microvascular areas (%) were measured from CD31 or CD34 immunostained sections at ×40 magnification. Slides were selected from injection and non-injection regions (apical and cranial from injection regions). “Whole heart” measurement was performed, including all the selected fields of injection and non-injection regions (apical and cranial from injection regions). All measurements (excluding macrovessels) were performed with Fiji ImageJ2 software in a blinded manner from four different fields (three left ventricular fields and one right ventricular field) of randomly selected tissue sections. Recruited immune cell (polymorphonuclear myeloid-derived suppressor cell) counts were analyzed from Ly6G immunostained sections at ×10 magnification. In addition, endothelial progenitor cell (EPC) recruitment into cardiac tissue was confirmed by triple immunostaining with CD31 (1:200, Abcam, ab28364), CD34 (1:200, Bio-Rad, MCA 1825), and CD117 (R&D systems, AF1356) antibodies or alternatively with VEGFR2 (1:200, Santa Cruz Biotechnology, sc-393163) antibody. Alexa fluor secondary antibodies: A488 chicken anti-rat secondary antibody (1:500, Invitrogen, A-21471), A594 chicken anti-rabbit secondary antibody (1:500, Invitrogen, A-21441), and A350 donkey anti-goat secondary antibody (1:500, Invitrogen, A-21081) or A350 goat anti-mouse secondary antibody (1:500, Invitrogen, A-21050) were used, respectively, and the slides were mounted with VECTAMOUNT AQ aqueous mounting medium (Vector Laboratories, H-5501-60).

### Multiplex Protein Level Measurement

Protein levels (VEGF-A, Angiopoietin-2, GM-CSF, M-CSF, G-CSF, IL-6, and TNF-α) from murine plasma or cell supernatants were analyzed on a single Luminex platform (R&D systems, LXSAMSM-07), according to the manufacturer’s instructions. In brief, samples were centrifuged at 16,000 × g for 4 min and diluted two-fold. Then 50 µL of each sample and standard were combined with 50 µL of microparticle cocktail on a 96-well-plate and incubated for 2 h on a shaker at 800 rpm. Following washing, the plate was incubated for 1 h with a biotin-antibody cocktail. Finally, streptavidin-PE was added to each well, and the plate was incubated for 30 min on a shaker at 800 rpm. The plate was read using a Bio-Rad analyzer (Bio-Plex 200 system).

### Endothelial progenitor Cell Assay

Circulating EPCs were characterized as described previously (Asahara et al., 1999; Hattori et al., 2001; Rehman et al., 2003). In brief, murine peripheral blood mononuclear cells (PBMCs) were isolated on d6 using Ficoll density-gradient centrifugation (Cytiva, 17544602) of mouse blood (500 µL) through SepMate-15 tubes (Stemcell technologies). Freshly isolated PBMCs were cultured in EBM^TM^-2 Basal Medium (Lonza, CC-3156) supplemented with EGM^TM^-2 MV Microvascular Endothelial Cell Growth Medium SingleQuots^TM^ (Lonza, CC-4147) on fibronectin-gelatin coated 6-well plates, and their identity was verified by metabolic uptake of Dil-acetylated-LDL (Dil-Ac-LDL) after incubation with 10 μL/ml of human Dil-Ac-LDL (Invitrogen, L3484) for 4 h. Adherent cells were visualized by fluorescence microscopy (Nikon H550L microscope). Half of the growth medium of the remaining cultured cells was changed every 3–4 days for 2 weeks. EPCs were further characterized by immunocytochemistry.

### Immunocytochemistry

Cultured EPCs on 18 × 18 mm glass coverslips were fixed with 4% PFA in PBS. After incubation at room temperature for 15 min, fixed cells were washed three times with PBS. Following washing, permeabilization was performed with 0.1% Triton X-100 (Fluka Analytical, 93418-250 ml) in PBS at room temperature for 10 min. Cells were incubated with primary antibodies [VEGF-A (Abcam, ab52917), VEGFR2 (Santa Cruz Biotechnology, sc-48161), and CD34 (1:200, Bio-Rad, MCA 1825)] for 1 h. After washes, cells were incubated with Alexa flour 488 and/or 594 secondary antibodies (Invitrogen). Samples were mounted on a slide grass with VECTASHIELD antifade mounting medium with DAPI (Vector Laboratories, H-1200-10) before fluorescence imaging using a Nikon H550L microscope.

### RNA Extraction and Quantitative RT-PCR Analyses

Approximately 30 mg of tissue samples were homogenized using Precellys 24 Tissue Homogenizer (Bertin Technologies SAS), and RNA was extracted using an RNeasy Fibrous Tissue mini kit (Qiagen, 74704) according to the manufacturer’s instructions. RNA from cultured cells was extracted using an RNeasy mini kit (Qiagen, 74106). One Microgram of total RNA was reverse transcribed into cDNA using random hexamers and RevertAID reverse transcriptase (Thermo Fisher Scientific, EP0441). Quantitative real-time PCR was performed using Powerup SYBR Green Master Mix (Applied Biosystems, A25741) and QuantStudio3 (Applied Biosystems) (Mushimiyimana et al., 2021) with the indicated primers shown in Supplementary Table S2. The real-time PCR data were analyzed using QuantStudio Software (Applied Biosystems). Results were calculated using the delta delta CT method (2^–∆∆CT^) (Schmittgen and Livak, 2001), with *HPRT* for normalization of *in vivo* samples and *GAPDH* for normalization of *in vitro* samples.

### Statistics and Reproducibility

No samples were excluded from the analysis. Unless otherwise indicated, experiments were replicated at least once for all analyses and the number of reproductions of each experimental finding is described in each figure legend. Data are presented as mean ± SD. Statistical differences between the means were compared by the two-tailed unpaired *t*-test for two groups or determined using one-way ANOVA followed by Dunnett’s multiple comparison test or two-way ANOVA followed by Sidak’s test for multiple groups unless otherwise noted. Statistical analysis was performed using Prism version 9 (GraphPad Software). Statistical significance was set to *p-*value <0.05 [*p* value style: **<**0.05 (*), **<**0.005 (**), **<**0.0005 (***), and **<**0.0001 (****)]. Non-significant *p* values were not mentioned.

## Results

### Proteolytically Resistant VEGF-B186R127S Has Different Receptor Binding Profile Compared to the Native Isoform

**FIGURE 1:**
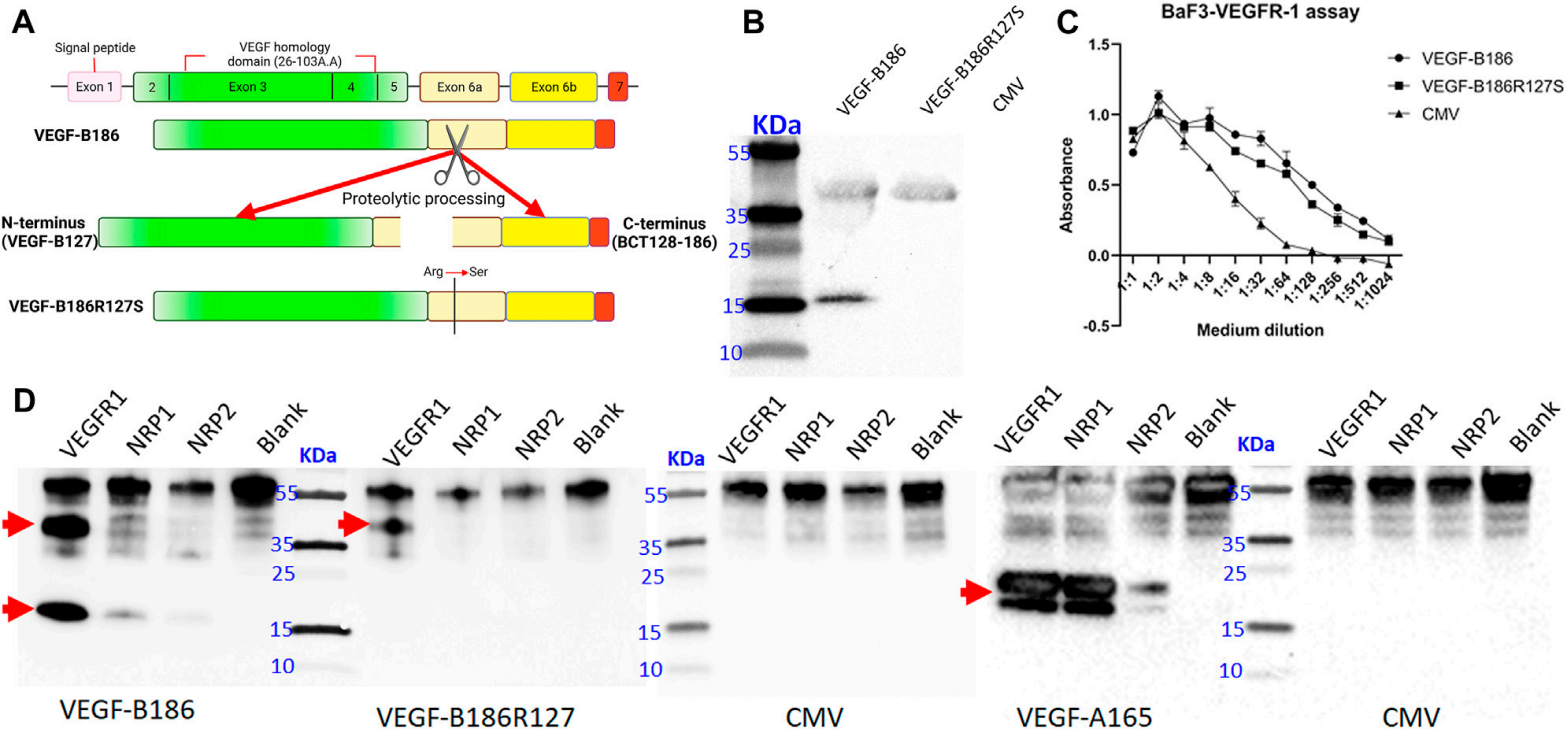
Characterization of the designed proteolytically resistant VEGF-B186R127S. **(A)** Graphical representation of VEGF-B186R127S. **(B)** Representative image of VEGF-B protein expression following HeLa cell transduction. **(C)** BaF3-VEGFR1 cell survival and proliferation assay. Each dot represents mean ± SD from two technical replicates from a representative experiment*.* **(D)** Receptor binding properties of different VEGF-B forms. Each lane represents the precipitation of ligands with hVEGFR1, hNRP1, hNRP2, or no receptor (blank). Red arrow signs indicate the full-length and proteolytically processed VEGF-B186, VEGF-B186R127S, or VEGF-A165.

VEGF-B186 gene contains seven exons (Olofsson et al., 1996). Like other VEGFs, VEGF-B186 contains a highly conserved VEGF homology domain (encoded by part of exons 3–4) (green colored) (Figure 1A). Light yellow, dark yellow, and red colors represent exons 6a, 6b, and 7, respectively. VEGF-B186 is proteolytically cleaved at amino acid position 127 (exon 6a encoding region). In HeLa cells, protein translation and secretion of VEGF-B186R127S and VEGF-B186 were efficient (Figure 1B). As expected, immunoblotting data showed two bands in the case of VEGF-B186 (full form and cleaved N-terminal form), while VEGF-186R127S size corresponded to the full-length form of VEGF-B186.

VEGF-B186R127S was also shown to be biologically active in BaF3-VEGFR1 cell proliferation and survival assay (Figure 1C). Receptor binding profile analysis verified that VEGF-B186R127S only binds to VEGFR1 (Figure 1D). Full-length form of VEGF-B186 binds to VEGFR1, and after proteolytical processing, it also binds to NRP1, as did VEGF-A165 (used as a positive control). In this study, we found that VEGF-B186 binds not only to NRP1 but also to NRP2 after proteolytical processing, as did VEGF-A165 (used as a positive control) (Gluzman-Poltorak et al., 2001).

### NRP Binding Is Dispensable for VEGF-B186R127S-Induced Microvascular Growth in the Heart

**FIGURE 2:**
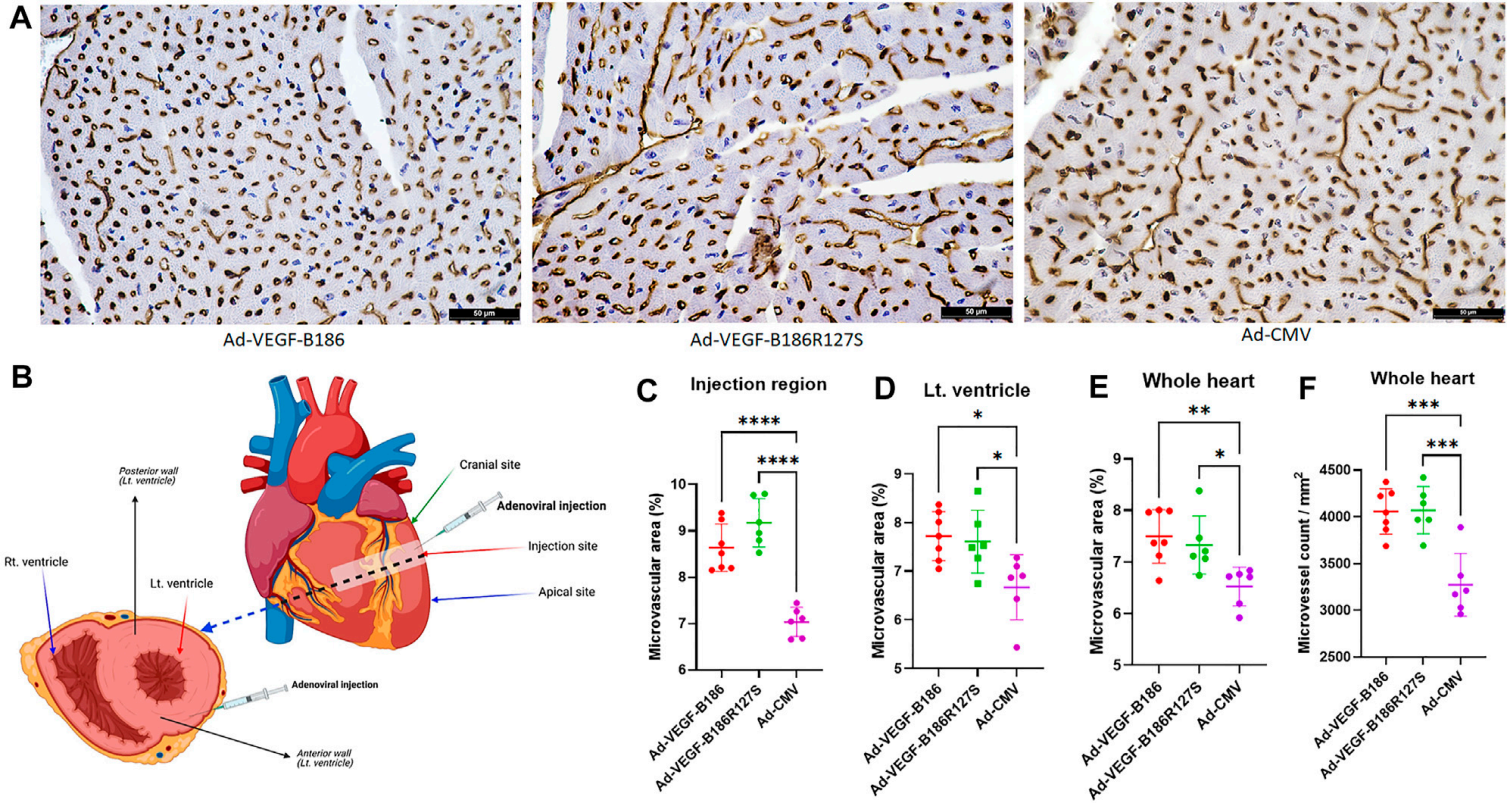
Myocardial vascular growth. (A) Representative images of CD31 stained heart tissue sections. Scale bars, 50 μm. **(B)** Diagram of the injected region and area of analyses. **(C–E)** Comparison of microvascular area in heart 6 days following gene transfer. **(F)** Comparison of the microvessel numbers in heart 6 days following gene transfer. Each dot indicates one mouse; *n* = 7 in Ad-VEGF-B186, *n* = 6 in Ad-VEGF-B186R127S, and *n* = 6 in Ad-CMV groups. Horizontal bars indicate mean ± SD and *p* values vs. each group by one-way ANOVA, followed by Dunnett’s multiple comparison test.

Microvascular growth was analyzed in mouse myocardium 6 days after the gene transfers. Total microvascular area was increased due to increases in capillary number and diameter. Overexpression of VEGF-B186R127S and VEGF-B186 led to significantly increased total microvascular area in the mouse heart (Figure 2). CD31 stained total microvascular area in the Ad-VEGF-B186R127S and Ad-VEGF-B186 injected regions was increased 1.3-fold compared to the control (Ad-CMV) (Figure 2C). When analyzing the left ventricle or the whole heart, Ad-VEGF-B186R127S and Ad-VEGF-B186 induced effects were increased approximately 1.2-fold (Figures 2D and E). We also analyzed microvascular counts to check if the microvascular area increases were due to new vessel formation and/or capillary enlargement. Compared to the control, microvessel counts were increased almost 1.2-fold in the Ad-VEGF-B186R127S and Ad-VEGF-B186 injected hearts (Figure 2F, Supplementary Figures S1A–C). In addition, the microvascular growth was associated with an enlargement of microvascular/capillary diameter (Figure 2A, Supplementary Figure S2A **).** Binding to NRPs was shown to be dispensable for microvascular growth since VEGF-B186R127S induced similar effects as native VEGF-B186.

Even though VEGF-B isoforms are secretory proteins, vascular growth occurred only close to the region of Ad-VEGF-B186R127S and Ad-VEGF-B186 injection areas. Thus, we did not observe any microvascular growth in apical or cranial sites of the left ventricle (Supplementary Figures S1D and E). As activated endothelial cells express CD34 (Goncharov et al., 2017), we also analyzed CD34-stained heart sections in the injected regions (Supplementary Figure S2A). CD34 stained total microvascular areas in the Ad-VEGF-B186R127S and Ad-VEGF-B186 injected regions were increased approximately 1.3-fold compared to the controls (Supplementary Figure S2B).

### VEGF-B186R127S Induces Endothelial Activation

**FIGURE 3:**
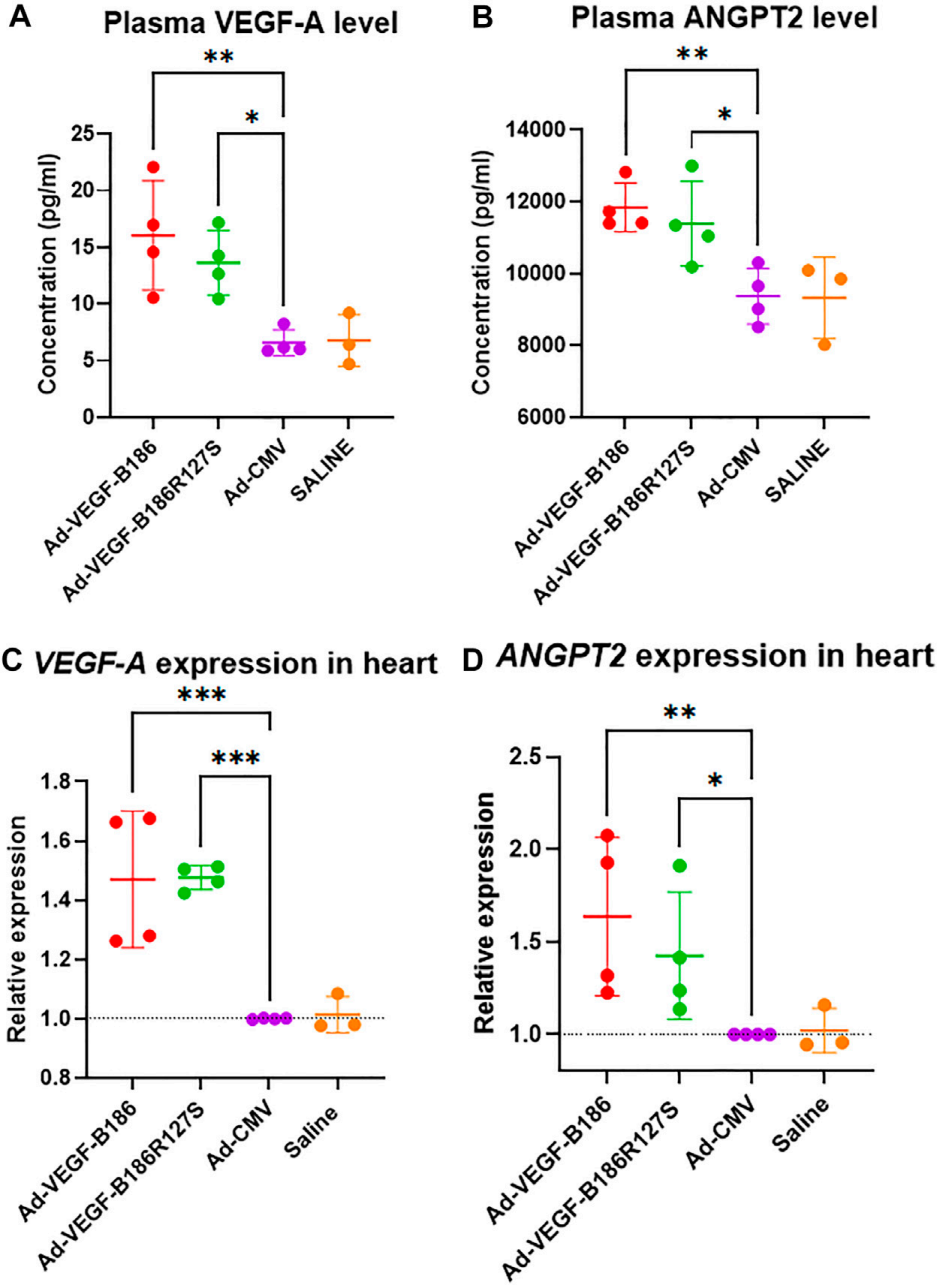
VEGF-B186R127S upregulates VEGF-A and ANGPT2 expression. **(A** and **B)** Comparison of plasma VEGF-A and ANGPT2 levels 6 days after gene transfer. **(C** and **D)** Comparison of *VEGF-A* and *ANGPT2* expression in heart 6 days following gene transfer. Each dot indicates one mouse; *n* = 4 in Ad-VEGF-B186, *n* = 4 in Ad-VEGF-B186R127S, *n* = 4 in Ad-CMV, and *n* = 3 in saline groups. Horizontal bars indicate mean ± SD and *p* values vs. each group by one-way ANOVA, followed by Dunnett’s multiple comparison test.

Angiopoietin-2 (ANGPT2) has been identified as a potent proangiogenic factor which functions together with VEGF-A during endothelial activation (Plank et al., 2004; Akwii et al., 2019). Intramyocardial Ad-VEGFB186R127S and Ad-VEGFB186 injections were shown to significantly upregulate VEGF-A and ANGPT2 levels in plasma 6 days following the gene transfers (Figures 3A and B). Similarly, *VEGF-A* and *ANGPT2* mRNA expression was significantly upregulated in heart muscle 6 days after the gene transfers (Figures 3C and D). We also verified significant upregulation of *VEGF-A* and *ANGPT2 in vitro* in Ad-VEGF-B186R127S and Ad-VEGF-B186 transduced cultured HMVEC-Cs (Supplementary Figures 3A and B). Thus, VEGF-B186R127S, similar to the native VEGF-B186 isoform, activates endothelial cells in the heart to contribute to microvascular growth.

### VEGF-B186R127S Induces Inflammatory Signaling

**FIGURE 4:**
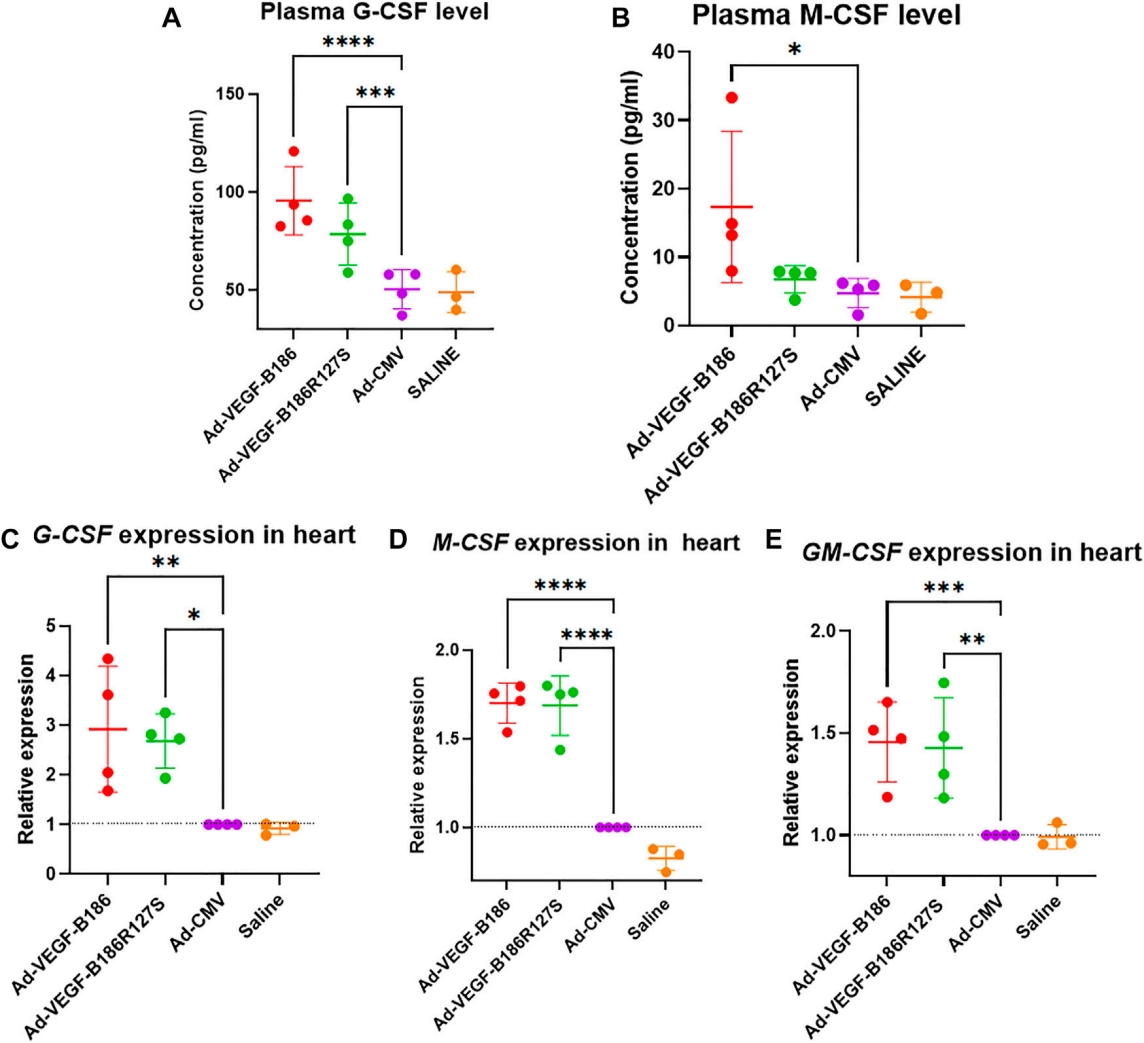
VEGF-B isoforms upregulate hematopoietic growth-inducing cytokine expression. **(A** and **B)** Comparison of plasma G-CSF and M-CSF levels 6 days after gene transfer. **(C–E)** Comparison of G-CSF, M-CSF and GM-CSF expression in heart 6 days following gene transfer. Each dot indicates one mouse; *n* = 4 in Ad-VEGF-B186, *n* = 4 in Ad-VEGF-B186R127S, *n* = 4 in Ad-CMV, and *n* = 3 in saline groups. Horizontal bars indicate mean ± SD and p values vs. each group by one-way ANOVA, followed by Dunnett’s multiple comparison test.

**FIGURE 5:**
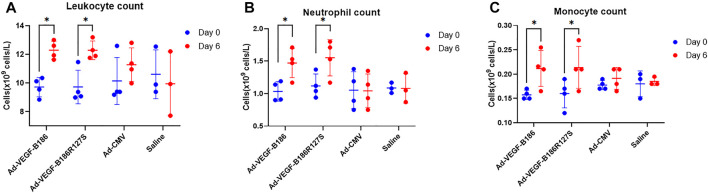
White blood cell analyses. **(A)** Comparison of leukocyte count from blood 6 days after gene transfer. **(B)** Comparison of neutrophil count from blood 6 days after gene transfer. **(C)** Comparison of monocyte count from blood 6 days after gene transfer. Each dot indicates one mouse; *n* = 4 in Ad-VEGF-B186, *n* = 4 in Ad-VEGF-B186R127S, *n* = 4 in Ad-CMV, and *n* = 3 in saline groups. Horizontal bars indicate mean ± SD and p values vs. each group by two-way ANOVA, followed by Sidak’s multiple comparison test.

**FIGURE 6:**
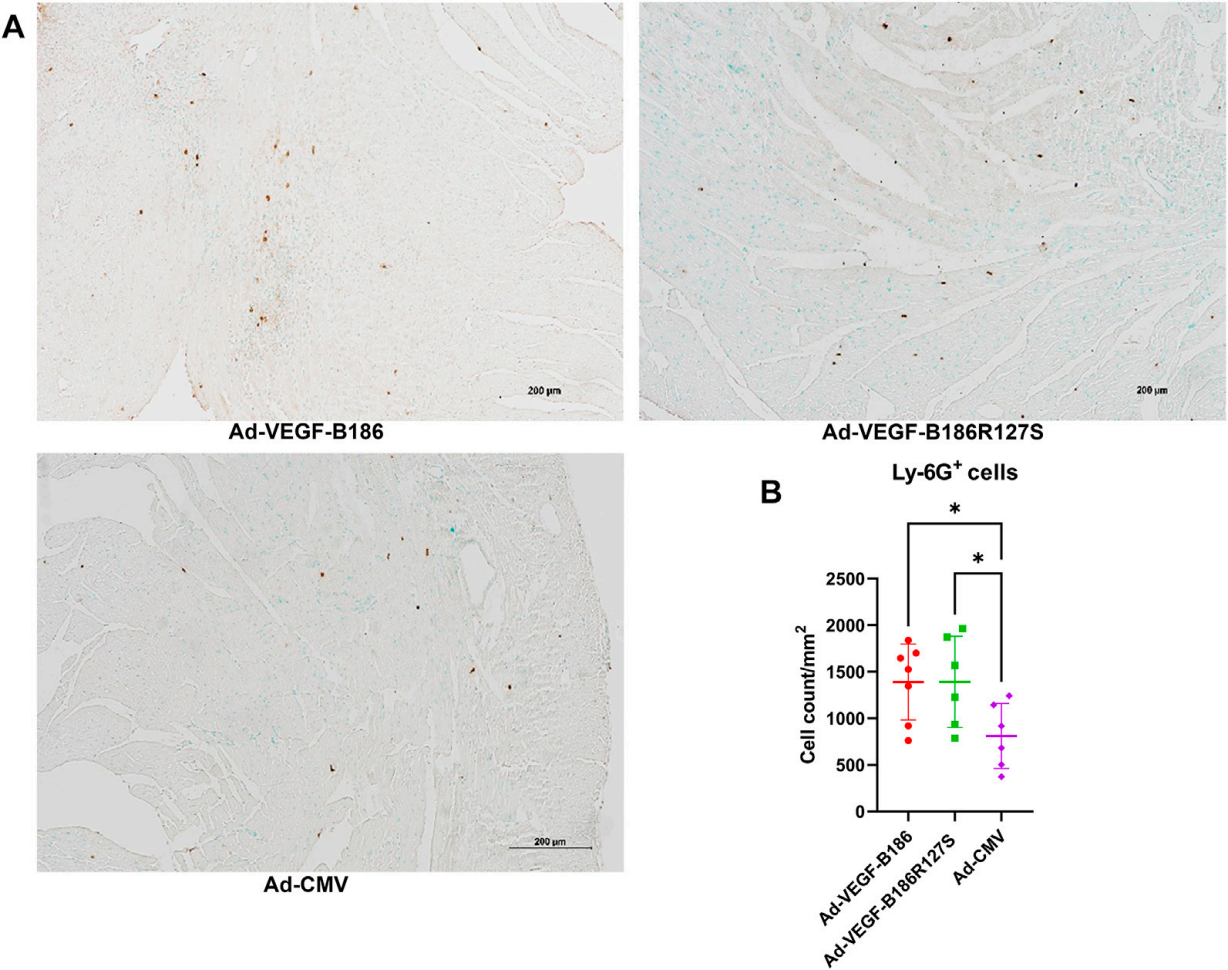
Local accumulation of Ly6G + cells in murine heart. **(A)** Representative image of Ly6G + stained heart tissue sections. Scale bars, 200 μm. (**B)** Comparison of Ly6G + stained microvascular area in adenoviral vector injected regions of heart. Each dot indicates one mouse; *n* = 7 in Ad-VEGF-B186, *n* = 6 in Ad-VEGF-B186R127S, and *n* = 6 in Ad-CMV groups. Horizontal bars indicate mean ± SD and *p* values vs. each group by one-way ANOVA, followed by Dunnett’s multiple comparison test.

We investigated other potential proinflammatory factors that are known to modulate vascular growth in the heart. Hematopoietic growth-inducing cytokines (G-CSF, GM-CSF, and M-CSF) are known to recruit and activate myeloid cells at the site of injury or inflammation (Metcalf, 2013). We found that intramyocardial Ad-VEGF-B186R127 significantly upregulated G-CSF levels, while Ad-VEGF-B186 injections upregulated both G-CSF and M-CSF levels in plasma 6 days following the gene transfers (Figures 4A and B). To confirm the source of these cytokines, we studied their expression in the heart tissue, where we found significant upregulation of *G-CSF, M-CSF,* and *GM-CSF* (Figures 4C–E). The number of circulating white blood cells, specifically neutrophils and monocytes, also increased (Figure 5). In addition, local accumulation of Ly-6G^+^ blood cells was shown to increase in the heart (Figure 6). Proinflammatory cytokines, such as TNF-α and IL-6, are also known to contribute to vessel growth (Fan et al., 2008; Prisco et al., 2016). We found that Ad-VEGF-B186R127S and Ad-VEGF-B186 injections significantly upregulated *TNF-α* in the heart tissue 6 days following the gene transfers, but IL-6 expression was not increased (Supplementary Figures S4A and B). We also studied *G-CSF*, *GM-CSF, M-CSF, TNF-α*, and *IL-6* mRNA expression *in vitro* (Supplementary Figures S3C–G). *G-CSF* and *M-CSF* expressions were significantly upregulated in VEGF-B186R127S and Ad-VEGF-B186 transduced HMVEC-Cs; however, *GM-CSF* expression did not show any changes (Supplementary Figures S3C–E). TNF-α and IL-6 expressions were significantly increased (Supplementary Figures S3F and G).

### VEGF-B186R127S Recruits Endothelial Progenitor Cells

**FIGURE 7:**
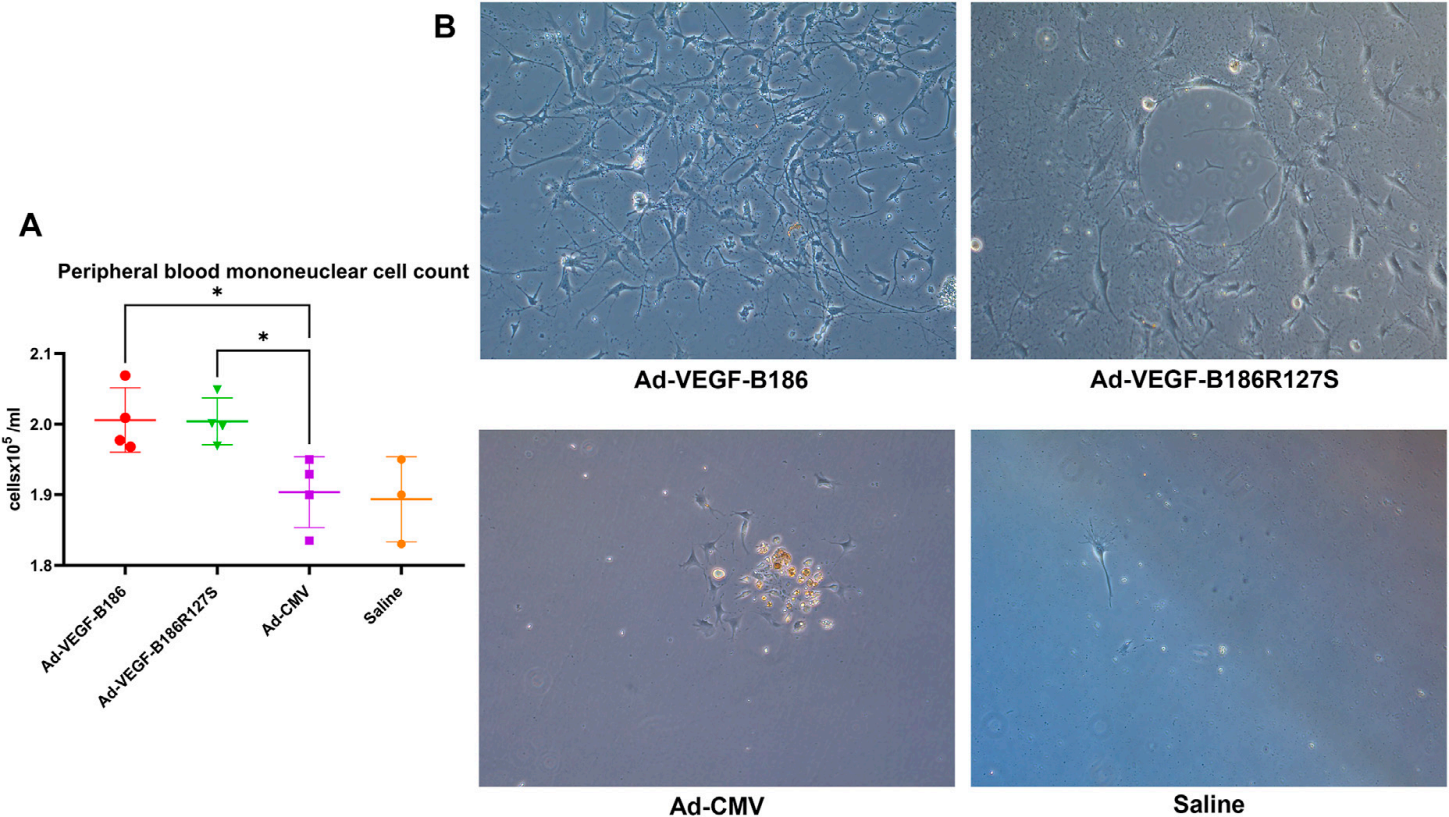
Endothelial progenitor cell growth from collected peripheral blood mononuclear cells. **(A)** Comparison of the number of circulating PBMCs 6 days after intramyocardial gene transfer. Each dot indicates one mouse; *n* = 4 in Ad-VEGF-B186, *n* = 4 in Ad-VEGF-B186R127S, *n* = 4 in Ad-CMV, and *n* = 3 in saline groups. Horizontal bars indicate mean ± SD and *p* values vs. each group by one-way ANOVA, followed by Dunnett’s multiple comparison test. (**B)** Representative images of cultured EPCs on culture day 14 (10× zoom).

**FIGURE 8:**
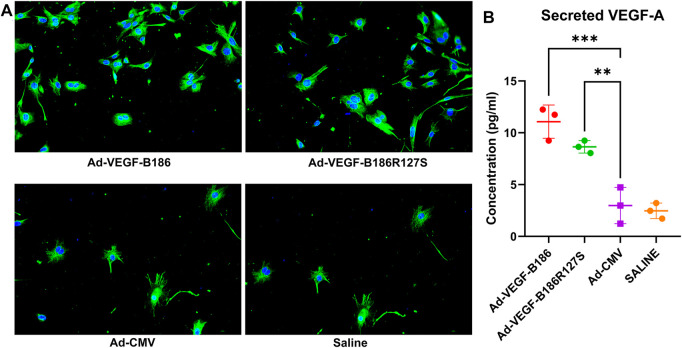
Endothelial progenitor cells secrete VEGF-A. **(A)** Representative images of VEGF-A (green) stained endothelial progenitor cells on culture day 15; Scale bars, 10 μm. **(B)** Quantification of VEGF-A concentrations from cultured EPC supernatants. Each dot indicates cultured EPC from one mouse; *n* = 3 in Ad-VEGF-B186, *n* = 3 in Ad-VEGF-B186R127S, *n* = 3 in Ad-CMV, and *n* = 3 in saline groups. Horizontal bars indicate mean ± SD and p values vs. each group by one-way ANOVA, followed by Dunnett’s multiple comparison test.

G-CSF and GM-CSF are potential chemotactic factors for immune cell recruitment along with EPCs (Kovacic et al., 2007). We found that the number of PBMCs was significantly higher 6 days after the intramyocardial Ad-VEGF-B186R127S and Ad-VEGF-B186 gene transfers (Figure 7A). As PBMCs are the potential source of EPCs, we verified the phenotype (by metabolic uptake of Dil-Ac-LDL; data not shown) and cultured the collected PBMCs in endothelial growth media for 2 weeks until complete EPC morphology was observed (Figure 7B). We characterized EPCs on day 15 by VEGFR2 and CD34 immunostainings (Supplementary Figure S5). We also found that EPCs were the potential source of VEGF-A secretion (Figure 8A). As Ad-VEGF-B186R127S and Ad-VEGF-B186 gene transfers significantly increased the number of circulating EPCs, the secreted VEGF-A concentrations were higher (Figure 8B). Since EPCs express CD34 (Yang et al., 2011), it also makes them possible mediators for the observed CD34-stained microvascular growth in Ad-VEGF-B186R127S and Ad-VEGF-B186 injected hearts (Supplementary Figures S2A and B). To verify the recruitment of EPCs into the heart, co-localization of CD31 and EPC markers such as CD34 and CD117 or VEGFR2 were demonstrated (Supplementary Figure S6).

### Cardiac function Is Unchanged After VEGF-B186R127S Gene Transfer

**FIGURE 9:**
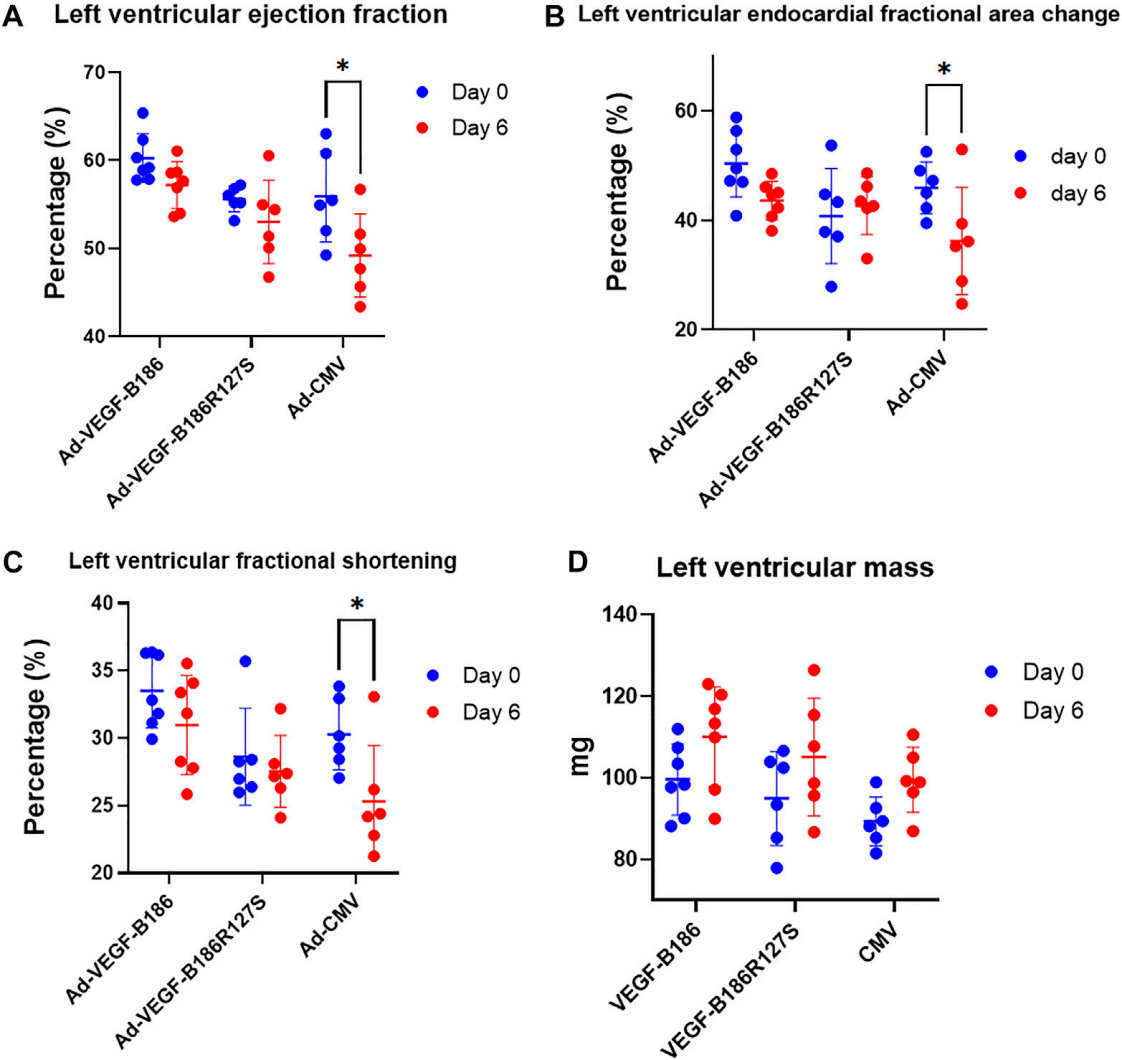
Cardiac function analysis. Analysis of left ventricular **(A)** ejection fraction, **(B)** fractional area change, **(C)** fractional shortening, and **(D)** left ventricular mass. Each dot indicates one mouse; *n* = 7 in Ad-VEGF-B186, *n* = 6 in Ad-VEGF-B186R127S, and *n* = 6 in Ad-CMV groups. Horizontal bars indicate mean ± SD and *p* values vs. each group by two-way ANOVA, followed by Sidak’s multiple comparison test.

Cardiac performance was closely monitored before and 6 days after intramyocardial injections. Despite having endothelial activation, intramyocardial Ad-VEGF-B186R127S and Ad-VEGF-B186 injections maintained unchanged left ventricular ejection fraction 6 days after the gene transfers (Figure 9A). Also, left ventricular fractional area change, fractional shortening, stroke volume, and left ventricular mass did not change 6 days after Ad-VEGF-B186R127S and Ad-VEGF-B186 gene transfers as compared to controls which showed impairment of the heart functions (Figures 9B–D) and no cardiac arrhythmias were detected in the study groups (Supplementary Figure S7).

## Discussion

In spite of the potential benefits of VEGF-B186, recent findings have indicated that VEGF-B186 gene transfer could cause ventricular arrhythmias, limiting the potential use of VEGF-B186 in cardiac gene therapy (Lähteenvuo et al., 2020). It has been suggested that VEGF-B186 binding to NRP1 after its proteolytical processing and consequent stimulation of sympathetic nerve growth is the mediator of these arrhythmogenic effects (Lähteenvuo et al., 2020). In the present study, we showed that the novel proteolytically resistant VEGF-B186R127S induced similar vascular growth in the mouse heart compared to the native VEGF-B186 without inducing arrhythmias. Since the maximal gene expression effect is visible 6 days after the gene transfer (Lähteenvuo et al., 2020), adverse reactions, such as arrhythmias, should be visible at the endpoint of our study. VEGF-B186R127S-induced vascular growth was shown to be independent on both NRP1 and NRP2 binding. This suggests that the full-length form of VEGF-B186 is responsible for mediating the angiogenic effects and NRPs are not needed in this process.

To explore the mechanism of VEGF-B186R127S induced angiogenesis, we focused on different proangiogenic factors. We observed the upregulation of VEGF-A, ANGPT2, and G-CSF proteins in plasma following intramyocardial Ad-VEGF-B186R127S and Ad-VEGF-B186 gene transfers. Similar upregulation was found in cardiac tissue samples in the corresponding mRNA levels. Hematopoietic growth factors, such as G-CSF and GM-CSF, are well-known to recruit EPCs and reduce local inflammation (Cho et al., 2003; Kovacic et al., 2007; Grote et al., 2011). EPCs are also known to overexpress VEGFR1 (Li et al., 2004), which supports the migration of EPCs toward the source of VEGF-B (cardiac endothelial cells). This is potentially another contributing factor for VEGF-B mediated angiogenesis. Circulating EPCs are the prognostic indicators for various cardiovascular diseases, including myocardial ischemic disease, and participate in angiogenesis post-infarction (Pirro et al., 2008; Chong et al., 2016; Kobayashi et al., 2017). Shintani *et al.* clinically demonstrated EPC mobilization to blood circulation following acute myocardial infarction (Shintani et al., 2001). In our study, the presence of circulating EPCs was also evident following Ad-VEGF-B186R127S and Ad-VEGF-B186 gene transfers. We also showed that EPCs produced high amounts of VEGF-A, which contributed to the upregulated plasma VEGF-A levels. Also, macrophage differentiating *M-CSF* expression levels were increased following intramyocardial Ad-VEGF-B186R127S and Ad-VEGF-B186 injections. The results correlated well with peripheral blood neutrophil and monocyte counts. We also verified the presence of Ly6G^+^ positive polymorphonuclear myeloid-derived suppressor cells as G-CSF, GM-CSF, and VEGF-A act as chemoattractants for these cells (Bronte et al., 2016; Dysthe and Parihar, 2020). Further studies would be needed to clarify the subtypes of white blood cells in the cardiac tissue following VEGF-B186R127S gene transfer. Overall, VEGF-B186R127S and VEGF-B186 induced signaling recruits EPCs and other immune cells into the heart contributing to the observed vascular growth.

Cardiomyocytes contribute around 70–85% of the heart volume, and the role of cardiac endothelial cells in maintaining vascular tone and angiogenesis and recruiting leukocytes and inflammatory cells into the heart is crucial (Shah, 1996; Pu et al., 2013; Zhou and Pu, 2016). Therefore, we also explored the mechanism of VEGF-B186R127S-induced angiogenesis at the cellular level in more detail. We showed that Ad-VEGF-B186R127S upregulated the expression of proangiogenic genes (*VEGF-A* and *ANGPT2*) and inflammatory cytokines (*G-CSF, M-CSF, TNF-α,* and *IL-6*) in human cardiac microvascular endothelial cells. Similar results were seen following Ad-VEGF-B186 transduction. Thus, endothelial cells release EPC-attracting growth factors, cytokines, and chemokines after transduction with Ad-VEGF-B186R127S (Summary).

VEGF-B-VEGFR1 mediated cardiac remodeling is crucial following myocardial infarction (Devaux et al., 2012; Räsänen et al., 2021; Tirronen et al., 2021). We found that Ad-VEGF-B186R127S and Ad-VEGF-B186 induced angiogenesis in the heart muscle while not hampering cardiac performance. VEGF-B186R127S induced similar effects as native VEGF-B186 in mouse heart without any side effects, such as cardiac arrhythmias. Our results indicate that the full-length VEGF-B186 is responsible for the angiogenic effects *via* cytokine-mediated EPC recruitment to the site of activated endothelium in the heart. The results support further development of VEGF-B186R127S gene therapy for clinical testing.

## Clinical Relevance

### What Is New?

• VEGF-B186R127S gene therapy causes microvascular growth in heart by recruiting endothelial progenitor cells.
• Proteolytically processed VEGF-B186 binds to neuropilin 1 and neuropilin 2, but there is no direct role of neuropilins in vascular growth stimulated by VEGF-B isoforms.

### Clinical Implications

• Insufficient perfusion (“no flow”) occurs frequently in ischemic and infarcted hearts due to microvascular obstruction in myocardial tissue.
• We showed that VEGF-B186R127S increases microvascular growth in the myocardium without causing arrhythmias.
• Designer VEGF-B186R127S gene therapy is a novel therapeutic approach to improve cardiac perfusion and provide structural and functional rescue of cardiac tissue in ischemic hearts.

## Data Availability

The raw data supporting the conclusion of this article will be made available by the authors, without undue reservation.

## Abbreviations

ANGPT2, angiopoietin-2; CMV, cytomegalovirus; DMEM, Dulbecco’s modified eagle’s medium; EPC, endothelial progenitor cell; d0, injection days; d6, sixth post-injection day; Dil-Ac-LDL, dil-acetylated-LDL; HMVEC-C, human cardiac microvascular endothelial cell; LVEF, left ventricular ejection fraction; LVEDV, left ventricular end–diastolic volume; LVESV, left ventricular end–systolic volume; NRP, neuropilin; PBMC, peripheral blood mononuclear cell; PBS, phosphate-buffered saline; PFA, paraformaldehyde; VEGF, vascular endothelial growth factor; VEGFR, vascular endothelial growth factor receptor; VP, viral particles.
