# Supplementary material for: Novel Designed Proteolytically Resistant VEGF-B186R127S Promotes Angiogenesis in Mouse Heart by Recruiting Endothelial Progenitor Cells
Source: Front Bioeng Biotechnol. 2022 Aug 4;10:907538. doi: 10.3389/fbioe.2022.907538 (PMC9385986; doi:10.3389/fbioe.2022.907538)
Supplement: Supplementary file 1 [file DataSheet1.docx]

Supplementary Material

## Supplementary Figures


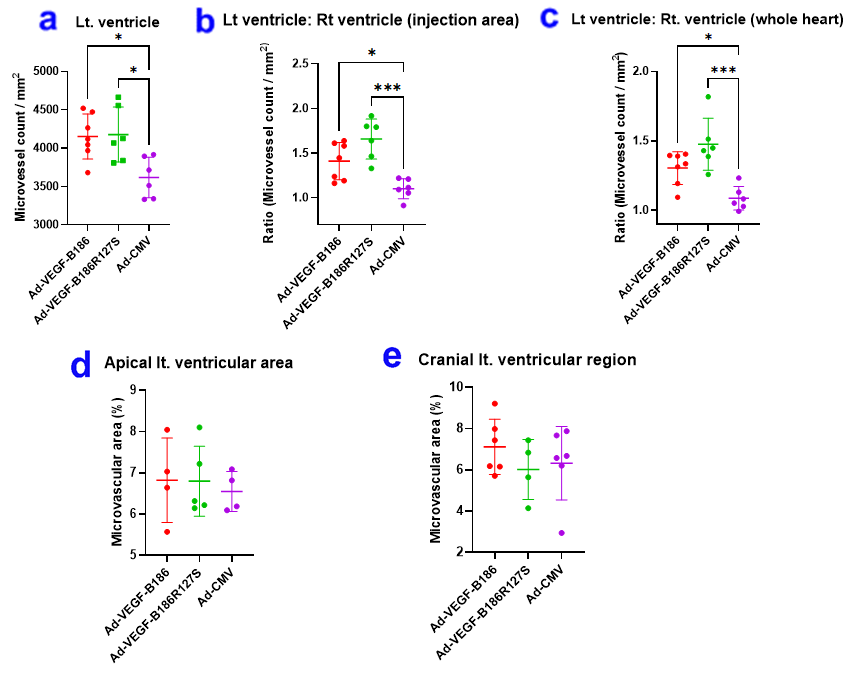


**Supplementary figure 1*. Analysis of microvascular growth from non-injected areas of heart***

**a** Comparison of microvessel numbers from left ventricle in heart. **b,c** Comparison of microvessel numbers ratio between left and right ventricles from injection sites and whole heart. Each dot indicates one mouse; n=7 in Ad-VEGF-B186, n=6 in Ad-VEGF-B186R127S, and n=6 in Ad-CMV groups. **d** Comparison of microvascular area from apical sites in heart. Each dot indicates one mouse; n=4 in Ad-VEGF-B186, n=5 in Ad-VEGF-B186R127S, and n=4 in Ad-CMV groups. **e** Comparison of microvascular area from cranial sites in heart. Each dot indicates one mouse; n=6 in Ad-VEGF-B186, n=4 in Ad-VEGF-B186R127S, and n=6 in Ad-CMV groups. Horizontal bar indicates mean ± SD and *P* values vs. each group by one-way ANOVA, followed by Dunnett’s multiple comparison test.


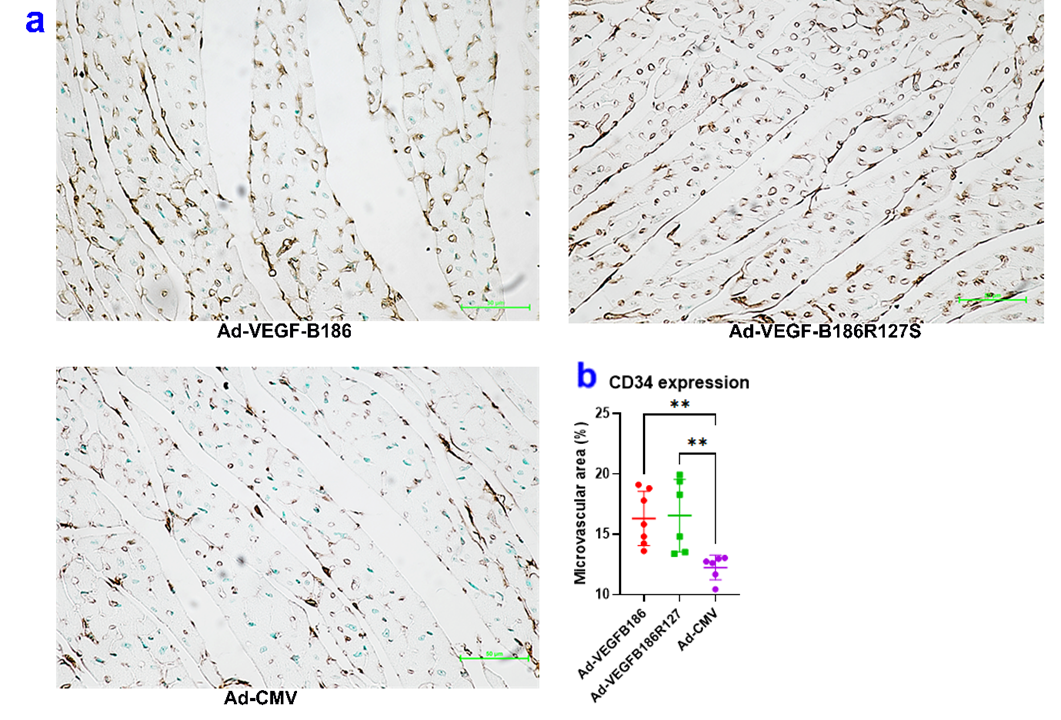


**Supplementary figure 2. *Activated endothelial cells in murine heart***

**a** Representative image of CD34 stained heart tissue sections. Scale bars, 50µm. **b** Comparison of CD34 stained microvascular area in adenoviral vector injected regions of heart. Each dot indicates one mouse; n=7 in Ad-VEGF-B186, n=6 in Ad-VEGF-B186R127S, and n=6 in Ad-CMV groups. Horizontal bars indicate mean ± SD and *P* values vs. each group by one-way ANOVA, followed by Dunnett’s multiple comparison test.


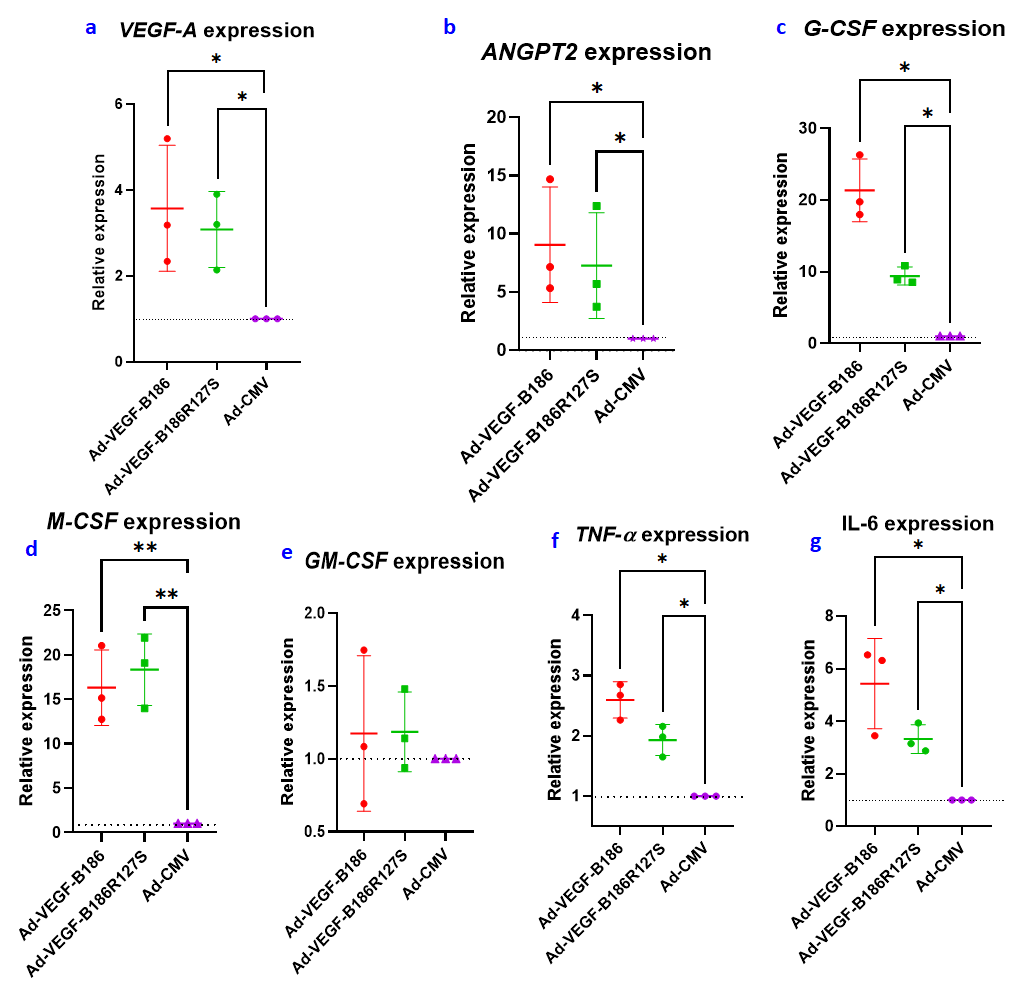


**Supplementary figure 3*. Contribution to neovascularization of cardiac microvascular endothelial cells***

**a-g** Comparison of *VEGF-A*, *ANGPT2*, *G-CSF*, *M-CSF, GM-CSF*, *TNF-α* and *IL-6* expression in Ad-VEGF-B186 (n=3), Ad-VEGF-B186R127S (n=3), and Ad-CMV (n=3) transduced HMVECs. Each dot indicates a mean of triplicate values from each independent experiment. Horizontal bars indicate mean ± SD and *P* values vs. each group by one-way ANOVA, followed by Dunnett’s multiple comparison test.


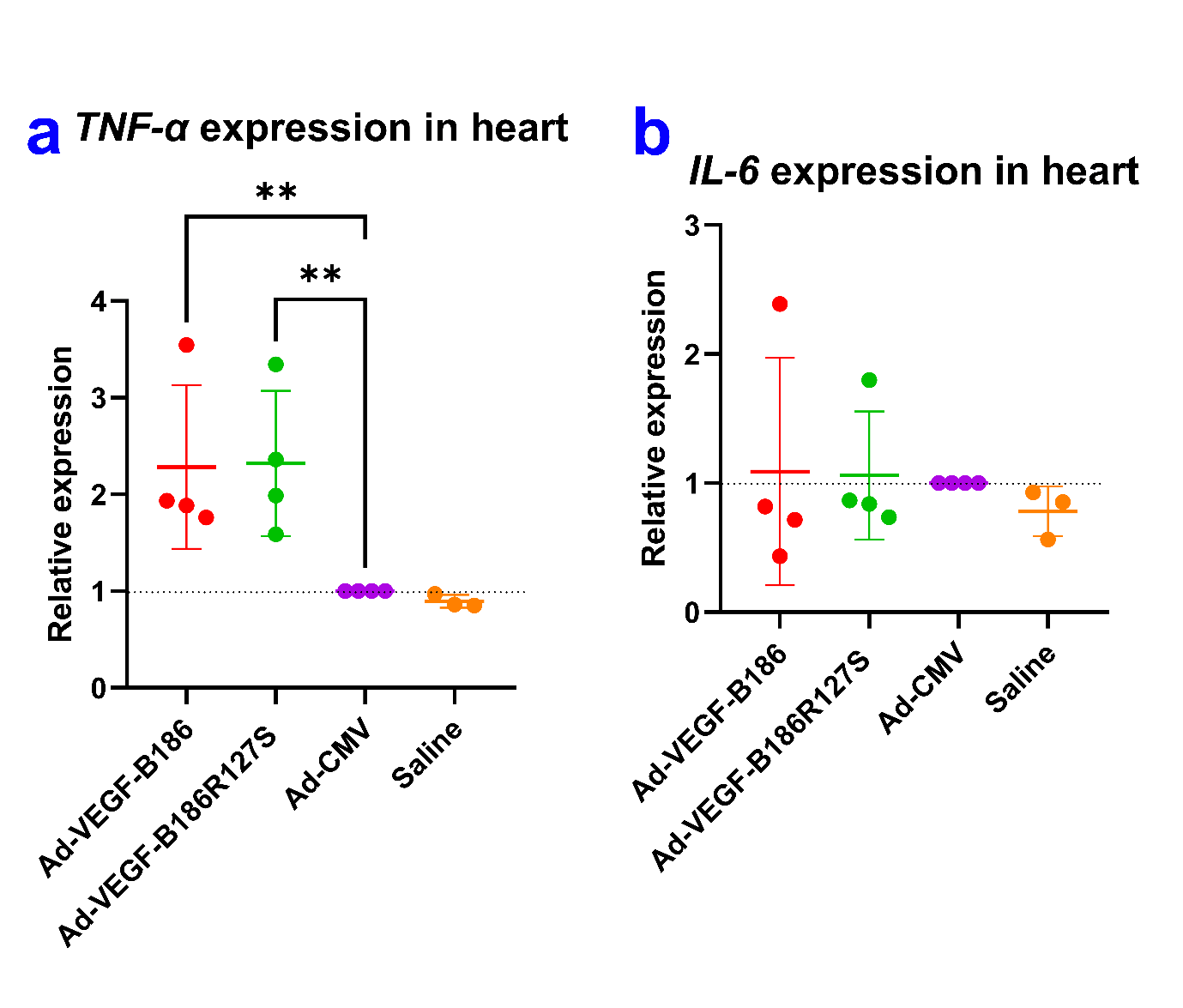


### Supplementary figure 4*. VEGF-B induced expression of inflammatory* *cytokines*

**a, b** Comparison of *TNF-α* and *IL-6* expression in heart 6 days after gene transfer. Each dot indicates one mouse; n=4 in Ad-VEGF-B186, n=4 in Ad-VEGF-B186R127S, n=4 in Ad-CMV, and n=3 in saline groups. Horizontal bars indicate mean ± SD and *P* values vs. each group by one-way ANOVA, followed by Dunnett’s multiple comparison test.

***
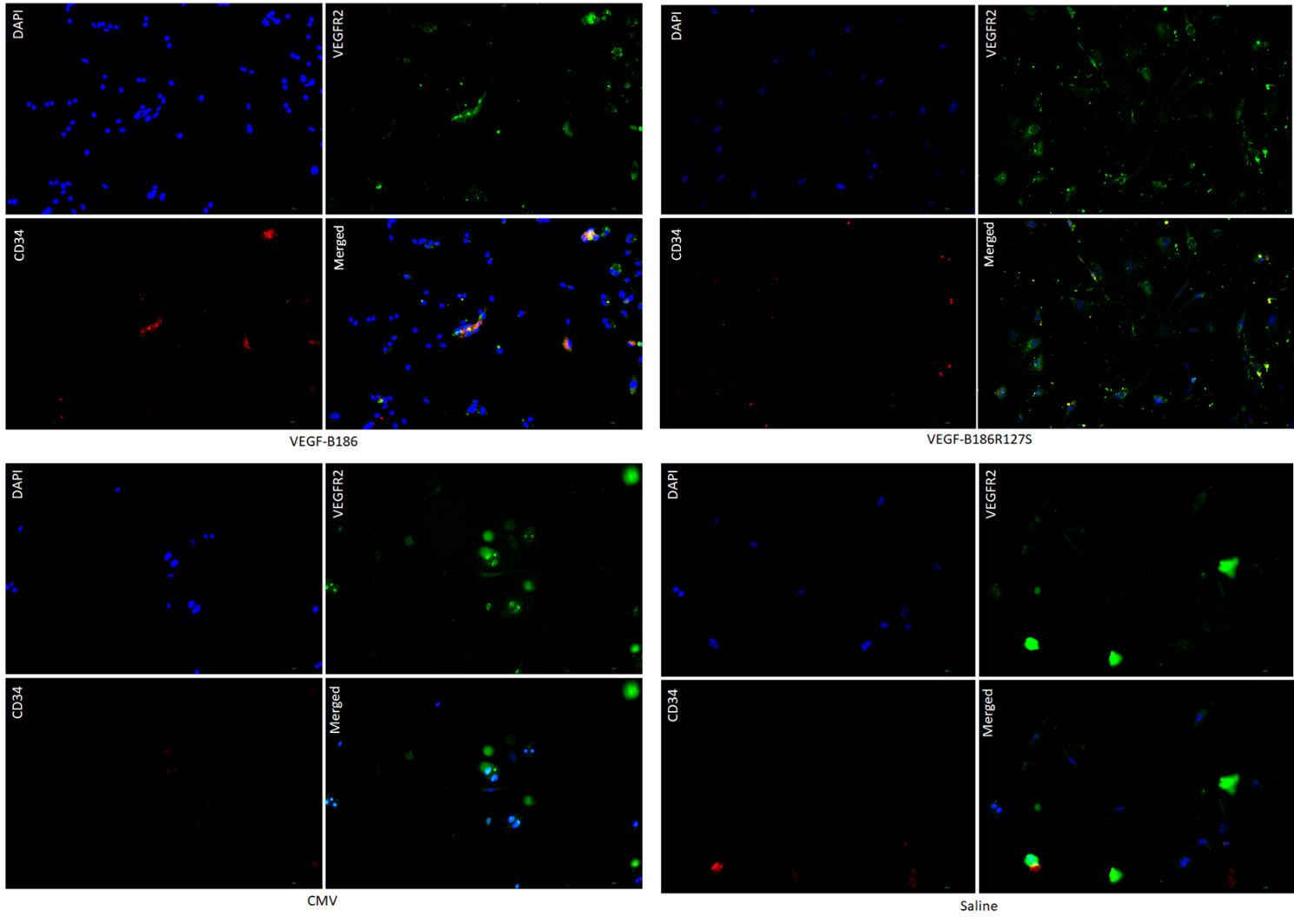
***

**Supplementary figure 5*. Characterization of endothelial progenitor cells***

Representative images of VEGFR2 (green) and CD34 (red) stained endothelial progenitor cells on 15^th^ day of culturing; Scale bars, 10µm.


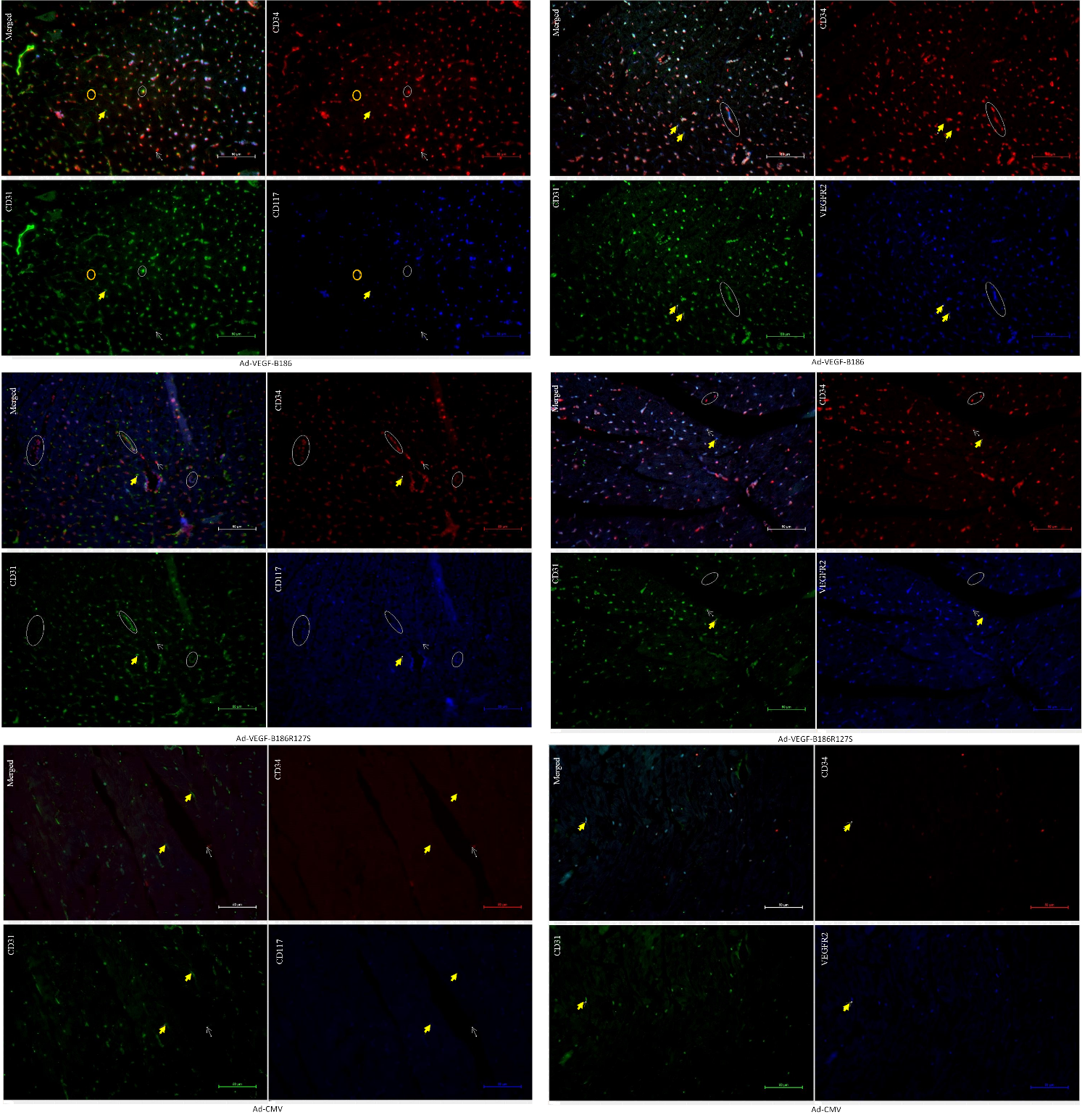
**Supplementary figure 6. *Recruited endothelial progenitor cell identification in murine heart***

Representative image of triple (CD31, CD34 and CD117 or VEGFR2) stained heart tissue sections. Scale bars, 50µm. White thin arrows and white ellipsoids indicate recruited endothelial progenitor cells (CD34 and CD117 or VEGFR2 staining) as well as activated endothelial cells (CD31, CD34 and VEGFR2 staining), while yellow thick arrows and yellow ellipsoids indicate existing endothelial cells or vessels (CD31 alone or combined with VEGFR2 staining).


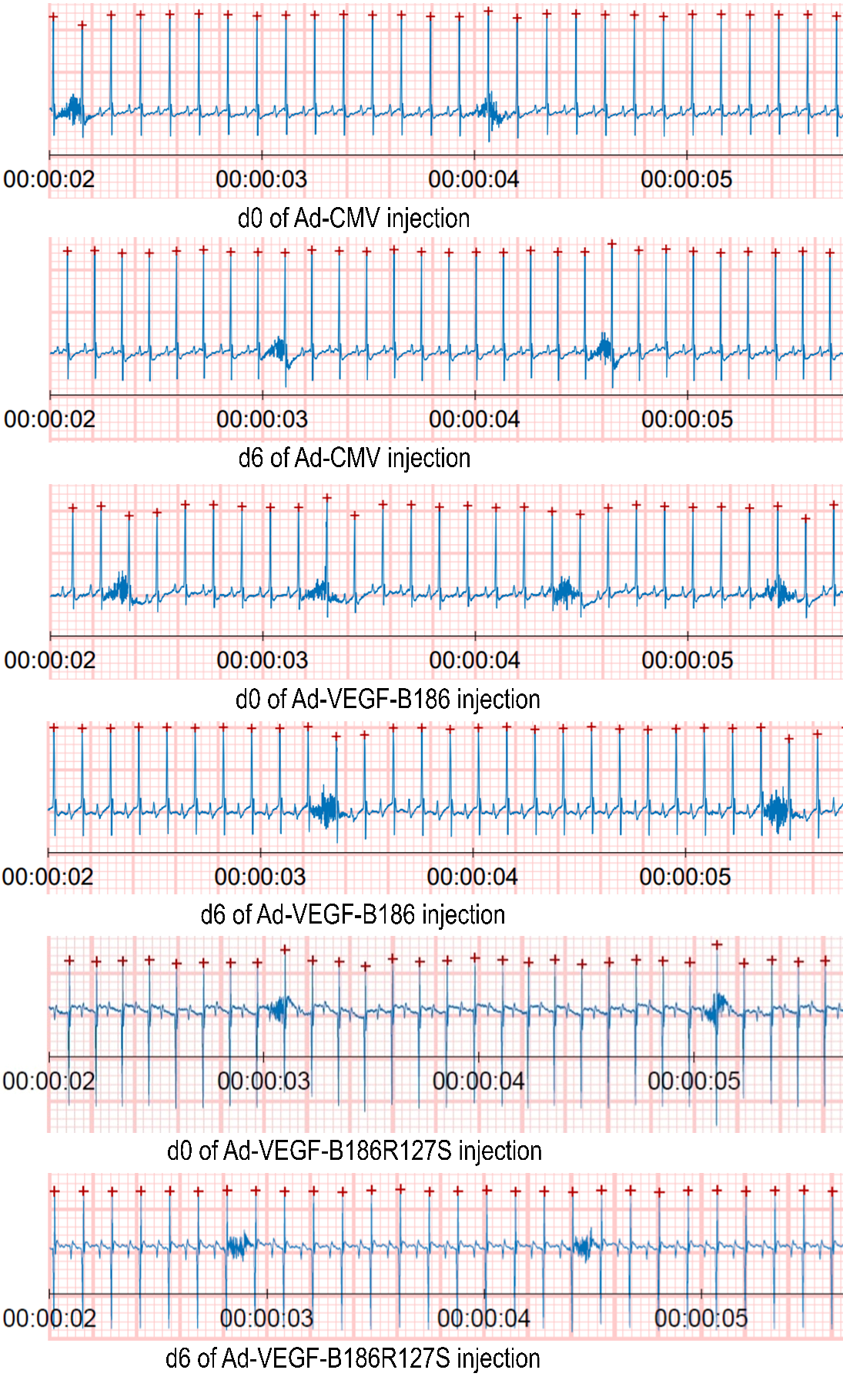


**Supplementary figure 7*. Electrocardiographic data analysis***

Representative images of electrocardiogram on d0 (before AdV injection) and d6. Electrocardiogram is recorded at speed of 25 mm/sec.

## Supplementary Tables

***Supplementary Table 1:*** *Study groups*

| **Group** | **Day 0** | **Day 6** | **Number of animals** | | | |
| --- | --- | --- | --- | --- | --- | --- |
| 1 | Gene transfer, LVEF, LVESV, LVEDV | LVEF, LVESV, LVEDV, and histology, | AdCMV | AdVEGF-B186 | AdVEGF-B186R127S | Saline |
|  |  |  | **6** | **7** | **6** | **-** |
| ***2*** | Gene transfer, differential count | differential count, PBMC isolation, EPC culturing, plasma protein data analysis, RT-PCR | AdCMV | AdVEGF-B186 | AdVEGF-B186R127S | Saline |
|  |  |  | **4** | **4** | **4** | **3** |

***Supplementary Table 2:*** *Primer sets for quantitative RT‐PCR*

| **Name** | **Sequence type** | **Strands** |
| --- | --- | --- |
| Mouse *ANGPT2* | Forward | CAGCAGCATGACCTAATGGA (Sense) |
|  | Reverse | ACAGTCTCTGAAGGTGGTTTG (AntiSense) |
| Mouse *CSF1 (M-CSF)* | Forward | GACAGATGAGAAGGAGCAGAAG (Sense) |
|  | Reverse | GCTGTCCCACCCTTTGAATA (AntiSense) |
| Mouse *CSF2 (GM-CSF)* | Forward | GTCACACAAATCAGGGACAGA (Sense) |
|  | Reverse | AGGAGCGTACTTGGCATTTAG (AntiSense) |
| Mouse *CSF3 (G-CSF)* | Forward | CATTCTCTCCACTTCCGAGTTT (Sense) |
|  | Reverse | AGCAGCAGCAGGAATCAATA (AntiSense) |
| Mouse *HPRT1* | Forward | GATCAGTCAACGGGGGACAT (Sense) |
|  | Reverse | GGGGCTGTACTGCTTAACCA (AntiSense) |
| Mouse *IL-6* | Forward | CTTCCATCCAGTTGCCTTCT (Sense) |
|  | Reverse | TTGGGAGTGGTATCCTCTGT (AntiSense) |
| Mouse *TNF-α* | Forward | CTACCTTGTTGCCTCCTCTTT (Sense) |
|  | Reverse | GAGCAGAGGTTCAGTGATGTAG (AntiSense) |
| Mouse *VEGF-A* | Forward | TGGTTCTTCACTCCCTCAAATC (Sense) |
|  | Reverse | CGGTCTCTCTCTCTCTTCCTT (AntiSense) |
| Human *ANGPT2* | Forward | ATCAGGACACACCACGAATG (Sense) |
|  | Reverse | CATCCTCACGTCGCTGAATAA (AntiSense) |
| Human *CSF1 (M-CSF)* | Forward | GGAGACCTCGTGCCAAATTA (Sense) |
|  | Reverse | CGCATGGTGTCCTCCATTAT (AntiSense) |
| Human *CSF2 (GM-CSF)* | Forward | GAGCTAGAAACTCAGGATGGTC (Sense) |
|  | Reverse | TCTTCTGCCATGCCTGTATC (AntiSense) |
| Human *CSF3 (G-CSF)* | Forward | TGTGTCCTTCCCTGCATTT (Sense) |
|  | Reverse | TTACCTATCTACCTCCCAGTCC (AntiSense) |
| Human *GAPDH* | Forward | GCAAGAGCACAAGAGGAAGA (Sense) |
|  | Reverse | CTACATGGCAACTGTGAGGAG (AntiSense) |
| Human *IL-6* | Forward | GGAGACTTGCCTGGTGAAA (Sense) |
|  | Reverse | CTGGCTTGTTCCTCACTACTC (AntiSense) |
| Human *TNF-α* | Forward | AGAGGGAGAGAAGCAACTACA (Sense) |
|  | Reverse | TGGGTCAGTATGTGAGAGGAA (AntiSense) |
| Human *VEGF-A* | Forward | GCTTACTCTCACCTGCTTCTG (Sense) |
|  | Reverse | CTGTCATGGGCTGCTTCTT (AntiSense) |
